# Multifaceted Actions of GFI1 and GFI1B in Hematopoietic Stem Cell Self-Renewal and Lineage Commitment

**DOI:** 10.3389/fgene.2020.591099

**Published:** 2020-10-26

**Authors:** Hugues Beauchemin, Tarik Möröy

**Affiliations:** ^1^Institut de recherches cliniques de Montréal, Montreal, QC, Canada; ^2^Division of Experimental Medicine, McGill University, Montreal, QC, Canada; ^3^Département de Microbiologie, Infectiologie et Immunologie, Université de Montréal, Montreal, QC, Canada

**Keywords:** GFI1, GFI1B, hemogenic epithelium, hematopoietic stem cells, transdifferentiation, hair cells

## Abstract

Growth factor independence 1 (GFI1) and the closely related protein GFI1B are small nuclear proteins that act as DNA binding transcriptional repressors. Both recognize the same consensus DNA binding motif *via* their C-terminal zinc finger domains and regulate the expression of their target genes by recruiting chromatin modifiers such as histone deacetylases (HDACs) and demethylases (LSD1) by using an N-terminal SNAG domain that comprises only 20 amino acids. The only region that is different between both proteins is the region that separates the zinc finger domains and the SNAG domain. Both proteins are co-expressed in hematopoietic stem cells (HSCs) and, to some extent, in multipotent progenitors (MPPs), but expression is specified as soon as early progenitors and show signs of lineage bias. While expression of GFI1 is maintained in lymphoid primed multipotent progenitors (LMPPs) that have the potential to differentiate into both myeloid and lymphoid cells, GFI1B expression is no longer detectable in these cells. By contrast, GFI1 expression is lost in megakaryocyte precursors (MKPs) and in megakaryocyte-erythrocyte progenitors (MEPs), which maintain a high level of GFI1B expression. Consequently, GFI1 drives myeloid and lymphoid differentiation and GFI1B drives the development of megakaryocytes, platelets, and erythrocytes. How such complementary cell type- and lineage-specific functions of GFI1 and GFI1B are maintained is still an unresolved question in particular since they share an almost identical structure and very similar biochemical modes of actions. The cell type-specific accessibility of GFI1/1B binding sites may explain the fact that very similar transcription factors can be responsible for very different transcriptional programming. An additional explanation comes from recent data showing that both proteins may have additional non-transcriptional functions. GFI1 interacts with a number of proteins involved in DNA repair and lack of GFI1 renders HSCs highly susceptible to DNA damage-induced death and restricts their proliferation. In contrast, GFI1B binds to proteins of the beta-catenin/Wnt signaling pathway and lack of GFI1B leads to an expansion of HSCs and MKPs, illustrating the different impact that GFI1 or GFI1B has on HSCs. In addition, GFI1 and GFI1B are required for endothelial cells to become the first blood cells during early murine development and are among those transcription factors needed to convert adult endothelial cells or fibroblasts into HSCs. This role of GFI1 and GFI1B bears high significance for the ongoing effort to generate hematopoietic stem and progenitor cells *de novo* for the autologous treatment of blood disorders such as leukemia and lymphoma.

## Introduction

### GFI1 and GFI1B as Transcription Factors

**Figure 1:**
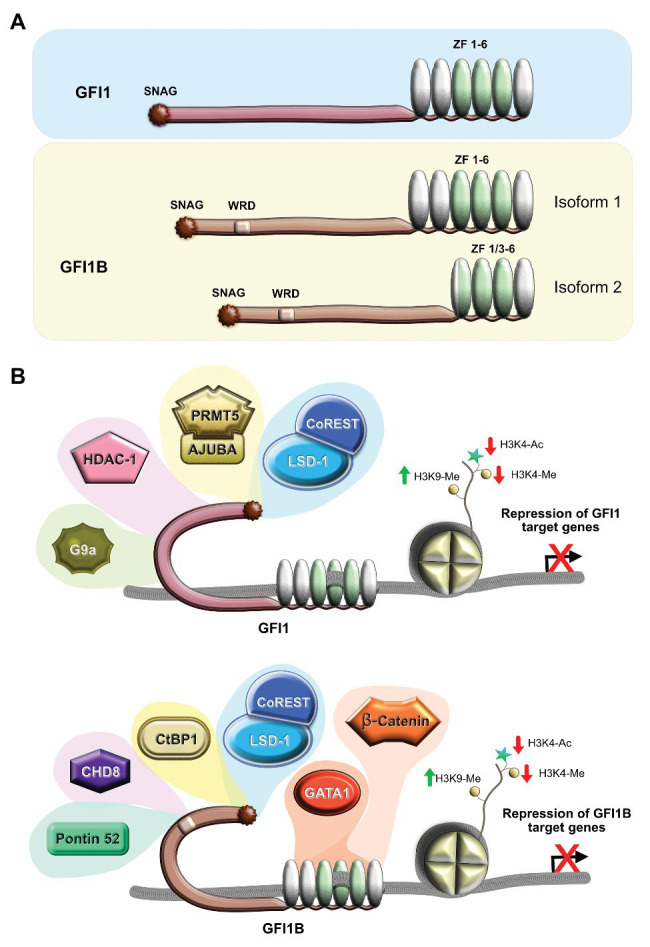
Structure and function of human GFI1 and GFI1B. **(A)** Schematic depiction of the structure of the proteins, showing the SNAG suppressor domain, the less characterized intermediate domain involved in protein/protein interactions, and the six zinc finger domains (ZF) localized at the C-terminal end with those three involved in DNA binding shown in green and the three other domains that play a role in interaction with other proteins shown in silver. The two isoforms of Gfi1b are shown with the longer megakaryocyte-specific isoform 1 that has the six zinc fingers and the short erythroid-specific isoform 2 that lacks two zinc fingers due to the fusion of ZF1 and ZF3. **(B)** Schematic representation of the GFI1 (top) and GFI1B (bottom) complexes with different partners that promote gene silencing by removal of open chromatin signatures and induction of marks that correlate with closed chromatin. WRD, Wnt regulatory domain.

The two members of the growth factor independence 1 transcription factor and proto-oncogene family, GFI1 and its paralogue GFI1B, are transcriptional repressors that play critical roles in hematopoiesis by regulating the cell fate of hematopoietic stem cells (HSCs), but also down the line in specific hematopoietic progenitor cells and precursors of more differentiated lineages. The *Gfi1* gene was first identified almost three decades ago in a screen for Moloney murine leukemia virus (Mo-MuLV) insertions as a factor promoting IL-2-independent growth in a mouse T cell lymphoma cell line (Gilks et al., 1993), whereas GFI1B was identified a few years later in human through a homology screening using low-stringency hybridization with the chick and the mouse *GFI1* zinc finger coding sequence (Rodel et al., 1998; Tong et al., 1998). Interestingly, the *Gfi1b* gene was also found later to be targeted by Mo-MuLV in c-Myc-dependent B-cell lymphomas in mouse (Mendrysa et al., 2010). Structurally, both GFI1 and GFI1B are constituted of three main domains that are very similar (Figure 1A). At the N-terminus of the proteins, there is a highly conserved SNAIL/GFI1 (SNAG) domain that is present in both GFI1 and GFI1B with ~90% homology, forming a sub-family on their own. This domain is shared with other transcriptional repressors such as SNAIL, SCRATCH, and SLUG forming a distinct but larger SNAG protein sub-family (Grimes et al., 1996; Manzanares et al., 2001; Katoh and Katoh, 2003, and reviewed in Chiang and Ayyanathan, 2013) and mediates transcriptional repression by recruiting chromatin modifier complexes to the regulatory regions of GFI1/GFI1B target genes (Tong et al., 1998; Saleque et al., 2007; Velinder et al., 2016; McClellan et al., 2019).

Both GFI1 and GFI1B share six zinc finger domains at their C-terminal ends, of which three (zinc fingers 3, 4, and 5) form the DNA binding domain that recognizes a sequence containing the core motif AATC [aAATCac(ta)gc for GFI1 and aAATCacaGc for GFI1B] (Lee et al., 2010; Maiques-Diaz et al., 2018b; Shooshtarizadeh et al., 2019; Vinyard et al., 2019), whereas zinc fingers 1, 2, and 6 mediate protein/protein interactions with factors such as MIZ-1 and PU.1 that can recruit GFI1 to target genes independently of DNA binding (Dahl et al., 2007; Basu et al., 2009; Liu et al., 2010). The proteins that similarly bind to the zinc fingers of GFI1B are less defined than those binding to GFI1, although we have identified β-catenin as a factor whose interaction with GFI1B depends on GFI1B’s zinc finger domain (Shooshtarizadeh et al., 2019) whereas GATA 1 has also been demonstrated to interact with this domain (Figure 1B; Huang et al., 2005; Rodriguez et al., 2005). Interestingly, in human, GFI1B, but not GFI1, exists as two splicing isoforms (Figure 1A), of which the smaller one lacks zinc fingers 1 and 2 but is still able to repress transcription with an efficiency similar to, or better than, the long, major isoform that has all six zinc fingers, suggesting that the first two are not essential for the repressive function (Laurent et al., 2012; Beauchemin et al., 2019). However, it has been shown that the two isoforms play distinct roles in hematopoiesis, with the long isoform being important for megakaryocyte differentiation and maturation and the short isoform regulating erythropoiesis, indicating that zinc fingers 1 and 2 of GFI1B holds a megakaryocyte-specific function, probably by binding yet-to-be identified megakaryocyte factor(s) (Laurent et al., 2012; Polfus et al., 2016; Rabbolini et al., 2017; Schulze et al., 2017). If both the SNAG domain and the zinc-finger domains are highly conserved between GFI1 and GFI1B, the two domains are separated by non-conserved intermediate domains whose function is not yet fully understood but have been shown to mediate protein-protein interactions with other factors such as the EHMT2 (G9a) histone methyltransferase, ATXN1 (Ataxin 1), PIAS3 and U2AF26 in the case of GFI1 (Rodel et al., 2000; Duan et al., 2005; Tsuda et al., 2005; Heyd et al., 2006; Andrade et al., 2016), or CHD8, CTBP1, and RUVBL1 (Pontin 52) through a six-amino-acid motif called Wnt regulatory domain (WRD) in the case of GFI1B (Shooshtarizadeh et al., 2019; Figure 1B).

The presently accepted model that describes the mode of action for both GFI1 and GFI1B is based on very robust biochemical evidence that both factors are able to recruit the histone demethylase LSD1, the histone methyltransferase G9a (in the case of Gfi1b also the methyltransferase SUV39H1), and the histone deacetylases (HDACs) or other enzymes with deacetylase activity such as Ajuba to target gene promoters or enhancers (Duan et al., 2005; Vassen et al., 2006; Saleque et al., 2007; Montoya-Durango et al., 2008). In the case of LSD1, the 20-amino-acid N-terminal SNAG domains of GFI1 and GFI1B are critical. This domain is in its sequence similar to the N-terminal end of the Histone H3 tail (Saleque et al., 2007; Chowdhury et al., 2013), and studies with the SNAG domain protein SNAIL1 have suggested that the affinity of the SNAG domain for LSD1 is higher than the histone H3 tail and therefore a binding of LSD1 to a SNAG transcription factor localized on chromatin is preferred over binding to histone H3 (Lin et al., 2010) and it is likely that the same is true for GFI1 and GFI1B. Transcriptional repression is achieved by the action of LSD1, which removes methyl groups from Histone H3 Lysine 4, thus contributing to a contraction of chromatin. HDACs that can bind to the domains of GFI1 and GFI1B located between the SNAG and zinc finger domains close accessibility to genomic sites by removing acetyl groups from histone H3 Lysine 9, thereby silencing transcription (Duan et al., 2005). The other histone-modifying enzymes, i.e., the methyl transferases G9a and SUV39H1 that also bind to regions of GFI1 or GFI1B located between the SNAG and the zinc finger domains, are part of this model as well. Both enzymes help to mediate the transcriptional repressor function of both GFI1 and GFI1B by catalyzing methylation of histone H3 Lysine 9 residues, which lead to contraction of chromatin or possibly even to the formation of transcriptionally inactive heterochromatin (Duan et al., 2005; Vassen et al., 2006).

However, parts of this model have been challenged by new findings using acute myeloid leukemia cells lines that indicated that the enzymatic activity of LSD1 as a demethylase is not per se required to mediate the function of GFI1, and by inference also of GFI1B, as transcriptional repressors. The study suggested that LSD1 functions rather as a scaffold protein enabling other enzymes such as HDACs to bind to GFI1 and GFIB and to act on histones in the vicinity of their target genes to silence transcription (Maiques-Diaz et al., 2018a,b). Evidence for this was provided among other data that an enzymatic inactive LSD1 can fulfill the same role as the wild-type enzyme. Future studies have to show how LSD1 exerts this scaffolding role, since it would suggest that HDACs associate with GFI1 and GFI1B indirectly *via* LSD1.

It is of interest to note that LSD1, which takes a central role in this model to mediate the transcriptional repressor function of GFI1 and GFI1B, has also other substrates than histones, one being the tumor suppressor and guardian of the genome p53. The p53 protein is methylated at lysine residues in its C-terminal regulatory region p53, which stabilizes it, restricts it to the nucleus, and enhances its ability to transcriptionally transactivate target genes (reviewed in Liu et al., 2019). LSD1 can remove these methyl groups and thus dampen p53 activity. In this context, a new function of GFI1 that is independent of its ability to bind DNA and act as a transcriptional regulator came to light. Proteome analyses revealed that GFI1 can bind to p53, and since both also bind to LSD1, a tripartite GFI1/p53/LSD1 complex is formed, which at least in lymphoid cells ensures the demethylation of the p53 C-terminal regulatory domain. In cells that lack GFI1, p53 remains methylated and is thus overactive and can induce apoptosis (Khandanpour et al., 2013; Vadnais et al., 2019). Similarly, GFI1 can also recruit other enzymes such as PRMT1 that methylate key DNA repair factors such as MRE11 or 53BP1 and regulate the response to DNA damage in this way (Vadnais et al., 2018). Similar abilities have not been reported for GFI1B, and it is thus likely that there may be major differences in molecular mechanisms that are employed by GFI1 versus GFI1B. Another difference between both factors may be the binding of the PIAS3 protein to GFI1 to affect the STAT3 signaling pathway, which has not been reported for GFI1B (Rodel et al., 2000). Moreover, the region in GFI1 that mediates the binding to PIAS3 contains a conserved type I SUMOylation consensus element, and recent reports suggest that SUMOylation of GFI1 favors the recruitment of LSD1 to target genes in hematopoietic cells (Andrade et al., 2016). By contrast, a mechanism that is employed by GFI1B, but for which no evidence exists that it may be also valid for GFI1, is the formation of a tripartite complex between GFI1B/β-catenin and LSD1. This complex occupies specific sites on chromatin and regulates β-catenin target genes in megakaryocytes, its precursors, and HSCs (Shooshtarizadeh et al., 2019). The fact that GFI1 and GFI1B are very similar proteins and in fact almost identical in sequence within their SNAG and zinc finger domains has raised the question how these factors can exert biological roles that are very different. The findings described in this last chapter and their very specific tissue and cell type-specific expression patterns provide first explanations for this conundrum, although more experimental data are needed to fully understand their differential regulatory potential.

Although both GFI1 and GFI1B seem to be strictly transcriptional repressors as there is no report published proposing a mechanism for an activating function, both of them are engaged in much more complex repression/activation loops due to the tight regulation of their expression either by themselves, as both GFI1 and GFI1B can repress their own expression, or from regulation through other hematopoietic master transcription factors such as RUNX1, GATA1/2, PU.1, and HMGB2 (Huang et al., 2005; Laurent et al., 2010; Wilson et al., 2010; Lancrin et al., 2012; Marneth et al., 2018). This tight regulation, characterized by waves of exclusive expression windows for GFI1 and GFI1B during the process of hematopoiesis from the emergence of hemogenic endothelial (HE) cells that occurs in the embryo to fully differentiated hematopoietic cells produced in adults, highlights a complex and multilayered function for these two key factors, which is discussed in depth in the next section.

## GFI1/GFI1B in Hematopoiesis and the Programming of HSCs

### From Endothelial-to-Hematopoietic Transition to Definitive Erythropoiesis

During the development, blood cells are produced in the embryo through three distinct waves called primitive, transient, and definitive hematopoiesis. During the first two waves of hematopoiesis that precede definitive hematopoiesis, blood cells are not derived from pluripotent HSCs, but rather from the conversion of special endothelial cells with hemogenic potential called HE cells or hemangioblasts that give rise to bipotent or multipotent progenitors (MPPs) that can colonize hematopoietic sites (reviewed in McGrath et al., 2015b; Gritz and Hirschi, 2016). The first wave that takes place as early as day e7.5 in the mouse is called primitive hematopoiesis and is often referred as to primitive erythropoiesis. The reason for this is the observation that this wave, which takes place in the blood islands of the yolk sac, produces essentially primitive nucleated erythrocytes and primitive megakaryocytes through conversion of hemangioblasts to primitive megakaryocyte-erythrocyte progenitors (MEPs) that are solely restricted to the erythroid and megakaryocyte lineages (Wong et al., 1986; Palis et al., 1999; Xu et al., 2001; Tober et al., 2007; Isern et al., 2011; Psaila et al., 2016).

The second wave that occurs shortly after the first, however, produces transient hematopoietic cells with an adult cell phenotype, or pro-definitive hematopoietic cells, including lymphoid cells. However, unlike cells produced during definitive hematopoiesis, those cells do not arise from single HSCs but rather from multipotent erythro-myeloid progenitors (EMPs) that seem to arise through hemogenic conversion of endothelial cells at different sites in the embryo such as vitelline, yolk sac, placenta, and the caudal intraembryonic splanchnopleura before colonizing the fetal liver (Godin et al., 1995; Cumano et al., 1996; Palis et al., 1999; McGrath et al., 2015a).

**Figure 2:**
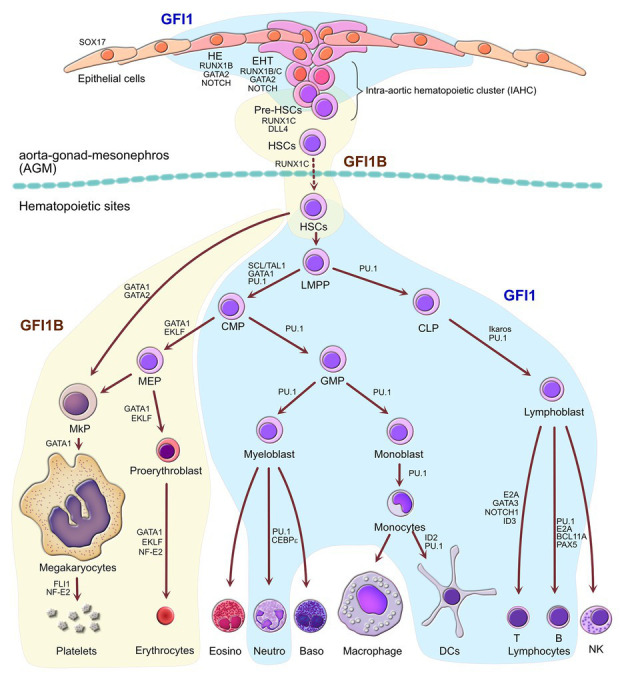
Separate roles of GFI1 and GFI1B during definitive hematopoiesis. Schematic representation of hematopoiesis from the initial production of primordial definitive hematopoietic stem cells (HSCs) from the hemogenic epithelial cells (HEs) found in the aorta-gonad-mesonephros (AGM) through endothelial-to-hematopoietic transition (EHT) during embryonic development until the production of all blood cells in the definitive hematopoietic sites such as fetal liver and adult bone marrow. Cellular compartments in which GFI1 plays a role are indicated as a blue area, whereas the compartment where GFI1B is important are indicated as the yellow areas, highlighting both overlapping and exclusive functions of GFI1 and GFI1B at all stages of hematopoiesis. Factors that contribute to cell fate decisions along with GFI1 or GFI1B are indicated. LMPP, lymphoid-primed multipotent progenitors; CMP, common myeloid progenitors; CLP, common lymphoid progenitors; GMP, granulocyte-monocyte progenitors; MEP, megakaryocyte-erythrocyte progenitors; MkP, megakaryocyte progenitors; Eosino, eosinophils; Neutro, neutrophils; Baso, basophils; DCs, dendritic cells; NK, natural killer cells.

Definitive hematopoiesis is the process by which all blood cells are produced from ultimately a pluripotent HSC with long-term repopulating capacity (LT-HSC). This process occurs through successive differentiation along a continuum of progenitor cells that acquire various molecular and cellular identity leading to diverse levels of commitment and biases toward specific lineages (reviewed in Brown et al., 2018; Laurenti and Gottgens, 2018). Those LT-HSCs first arise during a narrow window in development by a process called endothelial-to-hematopoietic transition (EHT). In this process, a specific subset of endothelial cells, the so-called HE cells located in the ventral wall of the aorta-gonad-mesonephros region (AGM), undergo a morphological change. During EHT, HE cells lose their endothelial traits and acquire a round, non-adherent shape that allows them to leave the AGM and colonize at first the fetal liver and later the bone marrow where they establish permanently (Figure 2; Christensen et al., 2004; Zeng et al., 2019, and reviewed in Dejana et al., 2017; Howell and Speck, 2020).

Early experiments had shown that both GFI1 and GFI1B play often reciprocal yet complementary roles during hematopoiesis (Figure 2) and this is mainly due to the regulatory feedback loop that exists between the two transcription factors that can actively repress the expression of each other (Doan et al., 2004; Vassen et al., 2005, 2007; Anguita et al., 2010). GFI1, which is normally expressed at readily detectable levels in HSCs and is critical for the maintenance and proliferative control of this population (Hock et al., 2004; Zeng et al., 2004), sees its expression subsequently increased in MPP cells where it favors cells to engage into the lymphoid lineage, although its expression is sustained in both the lymphoid and more mature myeloid precursor cells such as the granulocyte-monocyte progenitors (GMPs). In GMPs, *GFI1* expression drives the development of neutrophils by antagonizing eosinophilic differentiation, while expression of *GFI1* also ceases in cells differentiating into the monocyte-macrophage lineage (Figure 2; Hock et al., 2003; Dahl et al., 2007; Spooner et al., 2009; Li et al., 2010; Liu and Dong, 2012; Phelan et al., 2013; Fraszczak et al., 2016; Zhang et al., 2019). *GFI1B* on the other hand is highly expressed already in the HSCs but sees its expression decrease in the MPP compartment where it is replaced by *GFI1* whose expression starts to increase (Osawa et al., 2002; Vassen et al., 2007). Interestingly, *GFI1B* remains highly expressed in MEPs and in megakaryocyte precursors (MKPs) as well as during terminal differentiation of megakaryocytes and erythrocytes (Figure 2; Laurent et al., 2009). Studies with knockout mice have demonstrated that GFI1B plays a critical role in the biology of these two lineages (Saleque et al., 2002; Randrianarison-Huetz et al., 2010) and is pretty much absent from other cells of the myeloid and lymphoid compartment with the exception of a subset of early B-cells and rare plasmacytoid dendritic cells (pDC) where it seems to play a part in the regulation of the VD(J) recombination by repressing the *Rag1* gene through repression of the transcription activator FOXO1 (Osawa et al., 2002; Vassen et al., 2007; Schulz et al., 2012; Chow et al., 2013). It is not clear yet if GFI1B could play some role in rare myeloid cells, but high expression of functional GFI1B not only in erythroid/megakaryocytic lineage but also in non-erythroid/megakaryocytic myeloid cells has been associated with both chronic and acute myeloid leukemias, suggesting that it can act as a proto-oncogene possibly by supporting cell survival in cells where it is not normally expressed (Elmaagacli et al., 2007; Vassen et al., 2009). Intriguingly, a somatic dominant-negative mutation D262N in the fourth zinc finger of GFI1B that decreases its function has been associated with malignant transformation in an acute myeloid leukemia patient. The study suggests that the D262N mutant GFI1B feeds back on the function of the wild-type, unmutated GFI1B with the consequence that myeloid differentiation of precursors is favored over erythroid differentiation resulting in higher cell survival in MDS patient samples, which would attribute a direct oncogenic role to this GF11B mutant. This oncogenic activity could be mediated by the *Spi1 (PU.1)*, which is a direct GFI1B target gene and normally repressed by GFI1B. The GFI1B D262N mutant however leads to an increased expression of PU.1, leading the authors of this study to propose that a GFI1B-SPI1 interaction represents a critical element in the emergence of AML from MDS (Anguita et al., 2016). Such an association was not observed with any of the germ-line dominant-negative mutations identified in GFI1B, which represents a discrepancy that remains to be investigated (Stevenson et al., 2013; Monteferrario et al., 2014; Kitamura et al., 2016; van Oorschot et al., 2019). Similarly, loss of heterozygosity leading to low expression of GFI1B was also associated with myeloid leukemic transformation, suggesting that at normal level in early myeloid progenitors, GFI1B may act as a tumor suppressor, possibly by driving differentiation toward the erythroid lineage (Thivakaran et al., 2018).

The function of GFI1B in the terminal differentiation of both the erythroid lineage and megakaryocytes is critical but seems contradictory to some extent. The loss of GFI1B in MEPs leads on one side to an arrest in erythroid development at the erythroblast stage and ultimately to erythropoiesis failure, but also on the other side to an expansion of megakaryocytes that are however incapable to produce platelets due to a defect in proplatelet formation (Foudi et al., 2014; Vassen et al., 2014; Beauchemin et al., 2017; Shooshtarizadeh et al., 2019). Interestingly, overexpression of GFI1B in CD34+ human stem cells also leads to defects in erythroid differentiation with failure to produce cells beyond the proerythroblast stage, suggesting that a tight regulation of expression is required during the final maturation stages of red blood cells (Osawa et al., 2002). This dual, yet contradictory function raises questions on the biological role of GFI1B in cell fate decisions in MEPs that should either go toward the erythroid or the megakaryocyte lineages, in particular since high expression of GFI1B is required for both lineages. This apparent contradiction may have found its explanation in the recent discovery that megakaryocytes could derive directly through differentiation of HSCs therefore bypassing a multipotent EMP stage (Sanjuan-Pla et al., 2013; Shin et al., 2014, and reviewed in Yang et al., 2019).

Despite their critical role in definitive hematopoiesis, a role of GFI1 or GFI1B in primitive hematopoiesis or even during the transient hematopoiesis is still uncertain, and although such a role has been described for both GFI1 and GFI1B homologues Gfi1aa and Gfi1b in zebrafish, a requirement for either GFI1 or GFI1B in primitive erythropoiesis in mammals has not yet been identified (Cooney et al., 2013; Andrade et al., 2016; Velinder et al., 2017; Moore et al., 2018). Although mice lacking GFI1B die in utero at around day e14.5 because of a defect in erythropoiesis, the embryos survive until then because nucleated primitive erythrocytes are still generated although exhibiting some cellular abnormalities, indicating that GFI1B is dispensable to some extent at this stage for proper erythropoiesis to occur (Saleque et al., 2002). However, the absence of enucleated definitive erythrocytes in *Gfi1b*-knockout mice suggests that despite a definitive-like erythropoiesis occurring without HSCs during the transient hematopoiesis, GFI1B is required at this developmental stage (Saleque et al., 2002). Importantly, the simultaneous inactivation of GFI1 and GFI1B leads to a more severe reduction in pre-definitive erythrocytes and even defects in primitive erythrocytes, demonstrating that, indeed, GFI1 and GFI1B do play a role in the early hematopoietic development stages, albeit not as critical as during definitive hematopoiesis (Lancrin et al., 2012).

The critical role of both GFI1 and GFI1B in hematopoiesis is not restricted to the differentiation of HSCs into the different blood cell lineages but is already present in the process that gives rise to the first HSCs in the AGM of the embryo (Figure 2; Lancrin et al., 2012). Indeed, both proteins play an essential role in the repression of the endothelial genes within HE cells that undergo EHT to become HSCs, and it has been shown that this repression occurs through recruitment of LSD1 (Thambyrajah et al., 2016). Interestingly, during this process, GFI1 and GFI1B again exhibit mutually exclusive yet complementary expression patterns and functions. Although the role of master regulator of this conversion of HE cells pertains to RUNX1, its first action is to directly drive the expression of the GFI1/GFI1B factors, and it has been shown that forced expression of both can compensate for the loss of RUNX1 in this process (Lancrin et al., 2012). Indeed, the expression of RUNX1 is followed almost immediately by an increase in GFI1 in a subset of arterial endothelial cells of the AGM that defines them as hemogenic, despite the fact that they retain a fully endothelial phenotype (Thambyrajah et al., 2016; Porcheri et al., 2020). A subset of these GFI1-positive HE cells are then recruited in a Notch/DL4-dependent manner to the nascent intra-aortic hematopoietic cluster (IAHC) where they then start to express KIT (c-Kit). This marks the beginning of the EHT process during which cells silence their endothelial program and acquire a hematopoietic gene signature (Porcheri et al., 2020).

As the hematopoietic program gains in strength, GFI1 expression is gradually replaced by GFI1B, which along with the expression of CD45, marks the appearance of pre-HSCs. These cells begin to form clusters prior to their release into the bloodstream to colonize the fetal liver (Thambyrajah et al., 2016; Zeng et al., 2019). Intriguingly, the loss of either GFI1 or GFI1B does not prevent formation of IAHCs or the release of HSCs as does the loss of RUNX1 (Okuda et al., 1996; Saleque et al., 2002; Hock et al., 2004; Zeng et al., 2004; Zhang et al., 2019), but the loss of both GFI1 and GFI1B simultaneously completely abrogates the EHT process and leads to failure to produce any HSCs despite the formation of HE cells and the capacity of these cells to generate hematopoietic cells *in vitro* (Lancrin et al., 2012; Thambyrajah et al., 2016). A potential explanation for this observation could be the capacity, though limited, of GFI1 and GFI1B to compensate for the loss of each other (Fiolka et al., 2006). Indeed, the presence of either GFI1 or GFI1B, albeit in sub-optimal level, might just be sufficient to trigger EHT and to promote the recruitment of those transforming HE cells to the IAHC.

### *In vitro* Hematopoietic Transdifferentiation From iPS/ES Cells or Non-stem Cells

Generating transplantable HSCs from either embryonic stem (ES) cells, induced pluripotent stem (iPS) cells, or even directly from somatic non-stem cells such as differentiated hematopoietic cells and fibroblasts still represents today one of the most important challenges in medicine. Because of the critical role of both GFI1 and GFI1B at all stages of hematopoiesis, including the earliest developmental stages, it is not surprising that both factors were used in attempts to drive hematopoietic differentiation *in vitro* from both multipotent and differentiated non-hematopoietic cells.

It is possible today to generate all hematopoietic cell types from iPSCs, including HSC-like cells that express CD34. Cells have even been generated through processes that recapitulate *in vitro* the EHT that takes place in the AGM as a way to ensure acquisition of stemness to produce pluripotent HSCs with the capacity to colonize hematopoietic sites. However, these cells still lack a robust capacity to engraft in a host (Wang et al., 2005; Lengerke et al., 2009; Doulatov et al., 2013; Riddell et al., 2014; Ruiz et al., 2019; Zhou et al., 2019). The first experiment that succeeds to produce engraftable HSCs from iPS cells achieved this by driving differentiation *in vivo* through teratoma formation, but even in this case, the efficiency was very low with less than 1–2% of engraftment and relied on complex procedures and cocktails of cytokines to achieve this (Amabile et al., 2013; Suzuki et al., 2013; Philipp et al., 2018). Other approaches using *in vitro* 3D culture systems produced HSCs with similar engraftment potential without the need to go through a teratoma stage in mouse (Shan et al., 2020). It was only recently that by overexpressing either the single fusion protein MLL-AF4 or a large panels of seven transcription factors (RUNX1, ERG, HOXA5, HOXA9, HOXA10, LCOR, and SP1) but not including GFI1/GFI1B that the differentiation of iPS cells into engraftable HSCs showed more promising results, despite an elevated risk of leukemic transformation (Sugimura et al., 2017; Tan et al., 2018).

**Figure 3:**
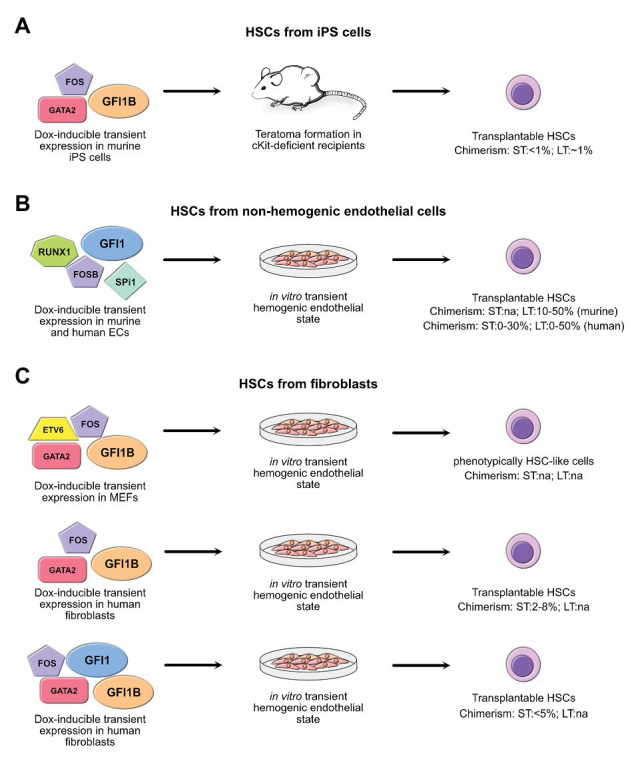
Summary of the strategies using overexpression of GFI1 and/or GFI1B. Shown are the combination of GFI1 and GFI1B with other factors to generate transplantable hematopoietic stem cells (HSCs) through trans-differentiation of non-hematopoietic cells, including induced pluripotent stem (iPS) cells **(A)**, non-hemogenic endothelial cells (ECs) **(B)**, and fibroblasts **(C)**. All these strategies involve an intermediary hemogenic endothelial state either *in vivo* by means of teratoma formation or *in vitro*. When known, the efficiency of HSC generation as assessed by measuring chimerism in graft assay in recipient mice is indicated for both short-term (ST) and long-term (LT) engraftment. na, not assessed. References: iPS cells (Tsukada et al., 2017); non-hemogenic endothelial cells (Sandler et al., 2014; Lis et al., 2017; Barcia Duran et al., 2018); fibroblasts (Pereira et al., 2013; Gomes et al., 2018; Daniel et al., 2019).

Using GFI1 or GFI1B to drive differentiation of iPS cells has been done only in one study by Tsukada et al. with moderate results (Tsukada et al., 2017). In this study, the authors drove expression of GFI1B together with FOS and GATA2 in murine iPS cells in a doxycycline-inducible way and were able to induce long-term HSC formation, but had to rely on teratoma formation to do so (Figure 3A). In their hands, forced expression of these sole three factors significantly improved the yield of engraftable HSCs that were produced compared to experiments that used unmodified iPS cells. Moreover, HSCs were obtained even without the need to co-inject supporting stromal cells along with the iPS cells or to provide hematopoietic cytokines, which are required to allow unmodified iPSC to transdifferentiate into putative HSCs (Amabile et al., 2013; Suzuki et al., 2013; Tsukada et al., 2017).

Clearly, the differentiation of iPS cells into transplantable HSCs is of high interest, but it does also carry its share of imponderable risks. In particular, teratoma formation remains a significant threat and takes place if cells with iPS-like properties remain after their differentiation into HSCs. For this reason, researchers have considered the possibility to produce HSCs through a direct conversion of differentiated cells, such as differentiated hematopoietic cells, endothelial cells, or even fibroblasts without passing through a pluripotent state. De-differentiation of committed hematopoietic progenitor cells into HSCs may seem to be the easiest way to achieve this, as a complete reprogramming, which is needed when more differentiated cells are used, is not necessary. Indeed, such a de-differentiation of hematopoietic progenitor cells has been achieved through combined overexpression of defined sets of transcription factors in both myeloid and lymphoid committed progenitors (Riddell et al., 2014). Conversion of endothelial cells into HSCs is also a natural approach as HSCs arise from HE cells and several protocols are already established for this, most of which use doxycycline-inducible transient overexpression of either Gfi1 or Gfi1b simultaneously with the overexpression of other factors such as GATA2, FOS, RUNX1, and SPI1 using lentiviral expression vectors (Figure 3B; Sandler et al., 2014; Lis et al., 2017; Barcia Duran et al., 2018; Daniel et al., 2019).

Conversion of fibroblasts into HSCs capable to produce mature definitive hematopoietic cells *in vitro* has also been achieved through the transient expression of the transcription factors Oct4 altogether with a cytokine treatment, or with transcription factor cocktails that include GATA2, RUNX1, LMO2, and ERG. However, the efficiency to produce cells with engraftable potential was low, and these methods resulted in cells that lacked the ability to produce lymphoid cells (Szabo et al., 2010) or lead to the production of HSCs with only short-term reconstitution ability (Batta et al., 2014). Other approaches have shown that transient expression of the transcription factors SCL, LMO2, RUNX1, and BMI1 can also differentiate fibroblasts into both lymphoid and myeloid progenitors capable of short-term engraftment in mice (Cheng et al., 2016). Interestingly, in this experiment, a key step into hemogenic conversion of fibroblasts was the upregulation of *Gfi1* controlled by LMO2 (Cheng et al., 2016). Importantly, generation of engraftable HSCs from fibroblasts was achieved by forcing transient expression of GFI1B in combination with GATA2 and FOS, either with or without ETV6, using pools of doxycycline-inducible lentiviruses (Pereira et al., 2013; Gomes et al., 2018). The efficiency of this approach was even further improved by adding GFI1 into the cocktail, allowing the generation of a similar number of hematopoietic stem-progenitor cells with short-term engraftable capacity in much shorter time, although long-term engraftment capacity was not assessed in this experiment (Figure 3C; Daniel et al., 2019). How GFI1/GFI1B forced transient expression contributes to the improvement of hemogenic conversion remains to be fully uncovered, but seems to be due to the capacity of at least GFI1B to directly cooperate with GATA2 by co-occupying promoters of critical non-hematopoietic genes leading to their silencing (Gomes et al., 2018). However, the lack of long-term hematopoietic reconstitution potential of cells from all these approaches demonstrates that despite a better understanding of hemogenic conversion, true reproduction of the initial generation of putative HSCs that occurs within the AGM during embryogenesis remains elusive.

### GFI1/GFI1B in Non-hematopoietic Cells

#### GFI1 in Ear Hair Cells

**Figure 4:**
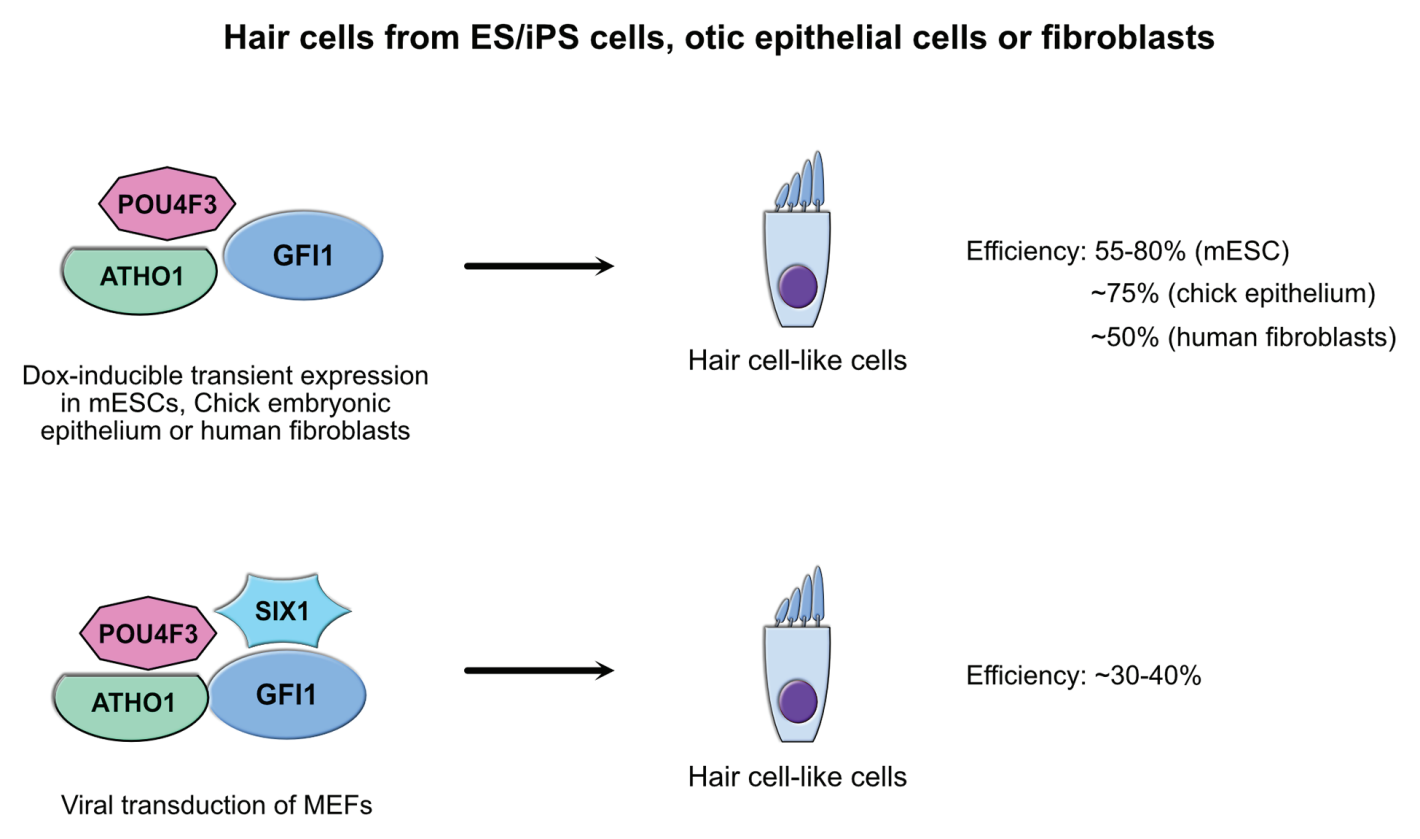
GFI1 and inner ear hair cells. Strategies to generate induced hair cell-like cells through trans-differentiation of non-hair cells *in vitro* by forced expression of GFI1 in combination with other factors. The efficiency to produce cells that harbor hair cell characteristics *in vitro* is indicated along the starting cell type used to achieve trans-differentiation (Costa and Henrique, 2015; Costa et al., 2015; Duran Alonso et al., 2018; Menendez et al., 2020).

Both GFI1 and GFI1B are prominently expressed in the hematopoietic system, but they are not entirely restricted to this compartment. Indeed, it has been shown that GFI1 for instance plays a critical role in the biogenesis of hair cells of the inner ear and that a loss of GFI1 leads to deafness and ataxia in mice (Wallis et al., 2003; Hertzano et al., 2004, and reviewed in Costa et al., 2017; Zhong et al., 2019). It has been reported that inner ear hair cells can be generated using forced transient expression of GFI1 in murine ES cells simultaneously with the transcription factor POU4F3 and its upstream hair cell master regulator ATHO1 using doxycycline-inducible polycistronic transgenes knocked into the HPRT locus to ensure balanced expression levels of all three factors (Hu et al., 2010; Masuda et al., 2011; Costa and Henrique, 2015; Costa et al., 2015; Figure 4). Likewise, direct conversion of otic epithelial cells from the inner ear into MYO7A-positive inner ear hair cell-like cells has also been achieved through simultaneous forced transient expression of GFI1, POU4F3, and ATOH1 in chicken embryos by *in ovo* electroporation of a similar doxycycline-inducible polycistronic transgene as the one used in mouse ES cells (Costa et al., 2015). Furthermore, recent experiments used transient overexpression of GFI1, ATHO1, and POU4F3, and included SIX1, a homeobox protein similar to *D. melanogaster* proteins called “sine oculis”, which is involved in autosomal dominant deafness type 23 (DFNA23) and has been shown to physically interact with the former three to target many hair cell regulators (Li et al., 2020). Either with or without SIX1, primary mouse embryonic and even adult human fibroblasts could be successfully converted into cells that exhibited most of the physiological traits of inner ear hair cells opening new avenues in potential treatment of deafness caused by the loss of these sensory cells (Figure 4; Duran Alonso et al., 2018; Menendez et al., 2020).

#### Gfi1 in the Pancreas

Besides the inner hair cells, GFI1 has also been suggested to play a role in pancreatic acinar cells, as one study has shown that its ablation results in defects in exocrine cells of the pancreas, characterized by a reduction of normal centroacinar cell markers and reduced exocrine function (Qu et al., 2015).

#### GFI1 in the Intestine

In addition, an ATOH1-dependent role of GFI1 in Paneth and mucus-secreting goblet cells of the intestine has also been suggested by the observation that the fate of these cells is shifted toward enteroendocrine cells in either *Gfi1*-knockout mice or *Atoh1*-knockout mice in which *Gfi1* expression is also turned off by the loss of ATOH1 (Shroyer et al., 2005; Bjerknes and Cheng, 2010; Gracz et al., 2018).

#### Gfi1b in the Intestine

GFI1 has been shown to be dispensable in intestinal tuft cells that do not depend on ATOH1 but mainly on POU2F3 for their survival and differentiation (Gerbe et al., 2011, 2016). These rare cells might be however more dependent on GFI1B instead, as they specifically express GFI1B at much higher levels than the GFI1-expressing cells that do not express GFI1B (Gracz et al., 2018; Wang et al., 2020). Although it is not known at this point whether expression of GFI1B is required for tuft cells or not, these cells are the only intestinal cells that express GFI1B in a POU2F3-dependent manner (Bjerknes et al., 2012; Huang et al., 2018; Wang et al., 2020). This was further supported by the observation that these two factors (GFI1B and POU2F3) are always co-expressed in tuft cells (Gerbe et al., 2016). This exclusive yet complementary expression pattern of GFI1 and GFI1B in intestinal endothelium cells is interesting as it replicates the specificity observed in the hematopoietic cells and demonstrates that this mutually exclusive expression regulation may be a typical feature of the GFI1 family of transcription factors.

## Conclusion

Producing engraftable HSCs from non-hematopoietic cells has represented the holy grail for many years and would mark a major breakthrough for clinical medicine. While the *in vitro* generation of almost all hematopoietic cell types is readily achievable to date, obtaining true engraftable HSCs remains elusive. It is of interest that GFI1 and GFI1B, two factors that are known to play important roles in all stages of hematopoiesis, including EHT during the initial production of definitive HSCs, might provide a way to generate those elusive HSCs. Although many obstacles have still to be overcome, considering the progress achieved during the past few years, it is conceivable that with the help of these two factors, a success in producing HSCs may be in reach in the foreseeable future. If such a breakthrough would be achieved, a number of new avenues would open up to treat a large spectrum of blood cell deficiencies as well as leukemia and lymphoma.

## Author Contributions

HB wrote text, collected references, and made the first version of the Figures. TM drew up the concept of the review, wrote text, and revised the Figures. Both the authors contributed to the article and approved the submitted version.

### Conflict of Interest

TM receives funding from the Canadian Institute of Health Research (CIHR, FDN-148372) and from a Canada Research Chair (CRC Tier 1) for work on GFI1 and GFI1B. Both are public Canadian funding organizations. TM acknowledges also funding from the Cancer Research Society (CRS), a Canadian Charity, for studies on Burkitt Lymphoma and from a VC fund (AmorChem) for work unrelated to the subject matter treated in this article.
